# White Matter and Neuroprotection in Alzheimer’s Dementia

**DOI:** 10.3390/molecules25030503

**Published:** 2020-01-23

**Authors:** Luca Lorenzini, Mercedes Fernandez, Vito Antonio Baldassarro, Andrea Bighinati, Alessandro Giuliani, Laura Calzà, Luciana Giardino

**Affiliations:** 1 Department of Veterinary Medical Sciences, University of Bologna, Ozzano Emilia, 40064 Bologna, Italy; luca.lorenzini8@unibo.it (L.L.); mercedes.fernandez@unibo.it (M.F.); andrea.bighinati@unibo.it (A.B.); a.giuliani@unibo.it (A.G.); 2 Interdepartmental Center for Industrial Research in Life Sciences and Technologies, University of Bologna, Ozzano Emilia, 40064 Bologna, Italy; laura.calza@unibo.it; 3 Department of Pharmacy and Biotechnology, University of Bologna, 40126 Bologna, Italy; vito.baldassarro2@unibo.it; 4 Fondazione IRET, Ozzano Emilia, 40064 Bologna, Italy

**Keywords:** amyloid, oligodendrocyte precursor cells, oxygen-glucose deprivation, drug screening

## Abstract

Myelin is the main component of the white matter of the central nervous system (CNS), allowing the proper electrical function of the neurons by ensheathing and insulating the axons. The extensive use of magnetic resonance imaging has highlighted the white matter alterations in Alzheimer’s dementia (AD) and other neurodegenerative diseases, alterations which are early, extended, and regionally selective. Given that the white matter turnover is considerable in the adulthood, and that myelin repair is currently recognized as being the only true reparative capability of the mature CNS, oligodendrocyte precursor cells (OPCs), the cells that differentiate in oligodendrocyte, responsible for myelin formation and repair, are regarded as a potential target for neuroprotection. In this review, several aspects of the OPC biology are reviewed. The histology and functional role of OPCs in the neurovascular-neuroglial unit as described in preclinical and clinical studies on AD is discussed, such as the OPC vulnerability to hypoxia-ischemia, neuroinflammation, and amyloid deposition. Finally, the position of OPCs in drug discovery strategies for dementia is discussed.

## 1. Introduction

“White matter” (WM) in the central nervous system (CNS) represents approximately 50% of brain mass [1]. WM mainly contains axons ensheathed by repeated windings of oligodendrocyte (OL) cytoplasm, providing a “myelin enriched” sheath which insulates the axons to permit proper electrical functioning of the neurons [2]. In fact, OLs are the cells responsible for myelin production in the CNS and contribute to providing metabolic support for the neurons.

The WM compartment is highly dynamic, due to the biochemical turnover of the main constitutive proteins (proteolipid protein (PLP) and myelin basic protein (MBP)) and lipids (cholesterol and the galactolipids galactosylceramide and sulfatide), but also to the phenomena known as “WM plasticity” or “myelin plasticity”. These terms refer to the de novo myelination of previously unmyelinated axons, to myelin sheaths replacement, or to the myelin remodeling which occurs during a person’s lifetime, as a consequence of multiple environmental factors such as voluntary physical exercise, social enrichment, motor learning, and cognitive training [3,4,5]. 

This plasticity, together with the increasing knowledge of the cell types responsible for myelin dynamics, i.e., the oligodendrocyte precursor cell (OPC), has raised a number of questions regarding the possible role of myelin and/or myelin-forming cells in neuropathology, not only in multiple sclerosis (MS), where the myelin is the target of the autoimmune attack, but also in a variety of other neurodegenerative diseases. 

In this review we will discuss data regarding the possible role of WM pathology in the onset/progression of Alzheimer’s dementia (AD), focusing on OPCs as potential therapeutic target.

## 2. White Matter Plasticity in the Adult Brain, Ageing, Cognitive Decline, and Alzheimer’s Dementia: Focus on Oligodendrocyte Precursor Cells (OPC)

An important observation highlighting WM contribution to the human brain function is that WM has expanded more than the grey matter throughout human evolution, suggesting that human cognition depends on brain connectivity [6,7]. The persistence of an appropriate myelin plasticity over the lifespan, moreover, means that axon wrapping by the OLs is modified depending on functional demand [8]: indeed the wrapping of OL cytoplasm around the axons determines the thickness of the myelin sheath and the internode length [9]. Moreover, OLs are metabolically active and functionally connected to the axon via cytoplasm-rich “myelinic channels”, through which the OL takes up blood-derived glucose and delivers glycolysis products (pyruvate/lactate) to myelinated axons via monocarboxylate transporters (MCT1 and MCT2) [10,11]. WM plasticity is influenced by a variety of regulators, including daylight rhythm, gender and pregnancy, voluntary physical exercise, environmental and social environment, motor learning, and cognitive training [3].

The cellular compartments most probably responsible for correlating complex behaviors to the myelin structure is the OL-axon unit. OLs derive from OPCs generated during development, which fully differentiate into myelinating OLs throughout birth and in the postnatal period. However, a significant population of OPCs persists in the adult CNS, as identified by the expression of the Neuron-glial antigen 2, a chondroitin sulfate proteoglycan referred as NG2 (CSPG4) and of platelet-derived growth factor alpha receptor (PDGFαR). Under appropriate conditions, resident and newly generated OPCs from neural stem cells (NSCs) of the subventricular zone (SVZ) actively proliferate, migrate to the nude axons, and differentiate into mature OLs [12,13]. NG2-positive OPCs are responsible for the generation of OLs across the lifespan to replace lost myelin, provide new OLs to myelinate new connections formed in response to increased neural activity, and repair the myelin in the event of injury or disease [14,15]. This process is finely and temporally regulated by a complex interplay between intrinsic and extrinsic factors.

OPCs also play an active role in activity-dependent neuronal plasticity, forming synaptic-like structures with presynaptic elements in several brain regions which contribute to activity-dependent neural plasticity [16,17], whereas presynaptic neurotransmitters bind to OPC receptors regulating OPC proliferation and differentiation [18,19]. Via this mechanism, therefore, which involves several molecules (ATP, LIF, adenosine, etc), receptors (NMDAR), and vesicular and non-vesicular release, neuronal inputs regulate OPC proliferation and differentiation [5]. Increasing recent evidence shows that neuronal activity induced by learning and by behavioral experiences such as locomotor activity promote myelination in vivo, whereas reduced social interaction decreases myelin-related gene products and myelin thickness [5]. This evidence obtained in laboratory animals is also supported by MRI studies in humans [20], proving the dynamic nature of myelin in adulthood and its close relation to neuronal activity.

Age-related structural and functional brain changes are well documented in humans as well as in laboratory animals, and include smaller global brain volumes, reduced cortical thickness, and expansion of the ventricular system, leading to the view that the decline in motor, sensory, and cognitive abilities in aging might be also associated with these structural changes [21]. The extensive use of MRI has shown that WM alterations in aging are numerous and extensive and different studies have identified WM atrophy [22], WM tract disruption [23], and loss of myelination [24]. Histopathological findings in human samples and aged experimental animals also reveal that the integrity of paranodes, which anchor the myelin sheath to the axon membrane, may be altered in aging, possibly due to the age-related alteration of MBP 21.5 kDa isoform, or the dysregulation of the enzyme cyclic nucleotide phosphodiesterase (CNPase). OL generation slows with ageing, due to the OPCs spending more time in the early G1 phase, resulting in a longer cell cycle [3]. OPCs in older mice also differentiate much more slowly than in younger mice, possibly because of epigenetic changes, i.e., histone deacetylase recruitment [25].

The contribution of WM alteration to the disturbance of cognitive function is documented by clinical neurology and MRI and Diffusion Tension Imaging (DTI), techniques which allow researchers to visualize the WM tract [7]. WM hyperintensities (WMHs) are related to cognitive dysfunction in the general population, and a significant WM loss manifesting both microstructurally and macrostructurally has been described in AD patients. These alterations are evident in several fiber pathways, while a reduction in fiber density and cross-section in mild cognitive impairment patients have been described in the posterior cingulum only. Notably, these degenerative changes have not been associated with a high amyloid plaque burden [26,27], while other studies correlates WM pathology in preclinical AD with biomarkers in the cerebrospinal fluid [28].

## 3. OPCs and Mature Oligodendrocytes in Alzheimer’s Disease and Animal Models

AD has classically been associated with a pathological Grey Matter (GM) process, where extracellular neuritic plaques of amyloid-beta (Aβ) and intraneuronal aggregates of neurofibrillary tangles made of phosphorylated tau protein are considered the main cause of neurodegeneration. In spite of the fact that, in the last years, many studies have demonstrated the involvement of WM in AD pathogenesis, the mechanism underlying these alterations, as also investigated in AD animal models, are not clear [29,30]. 

**Figure 1:**
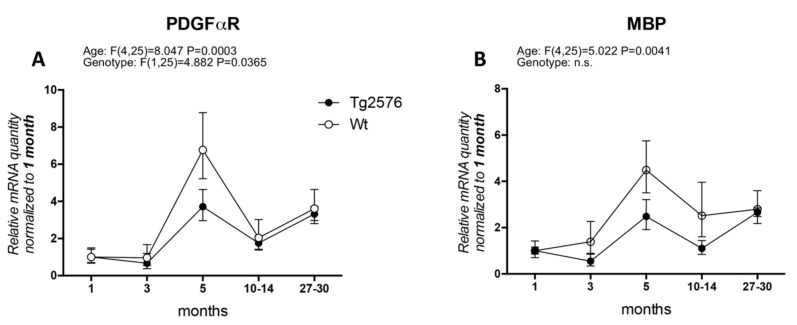
Age-related variation of oligodendrocyte lineage markers. (**A**) platelet-derived growth factor alpha receptor (*PDGFαR*) gene expression in Wt and Tg2576 at different age timepoints; (**B**) myelin basic protein (*MBP*) gene expression in Wt and Tg2576 at different age time points. Relative expression has been normalized to 1 month matched for each genotype. Statistical analysis has been performed through 2-way ANOVA, considering age (months) and genotype (Wt and Tg2576) as variables; n = 3–5. Results are significant when *p* < 0.05.

In particular, the timing of OPC and WM abnormalities with regard to the early onset of cognitive impairment has not been clarified. Actually, AD clinical signs appear after a presymptomatic preclinical phase lasting decades, which is also resumed by available animal models, and it is generally believed that this phase must be regarded with particular attention to find the appropriate time window for therapeutic approaches. We have extensively studied the histopathology and neurochemistry of the preclinical phase of AD in the mouse model Tg2576, carrying the APP KM670/671NL (Swedish) modification, showing that (1) intraneuronal and intraglial Aβ accumulation precedes amyloid plaque deposition [31,32]; (2) intraneuronal Aβ accumulation increases neuronal vulnerability to oxygen-glucose deprivation (OGD) [33]; (3) a molecular dysfunction involving HIF signaling in the cerebral cortex, also regarding the vascular endothelial growth factor receptor, is already present in 3-month-old animals, while plaque deposition starts at 8 months [32]; and that neurotransmitters (acetylcholine, GABA and glutamate) are also altered prior to plaque deposition [34,35]. We then investigated molecular markers for OPC and myelin formation at different ages (1, 3, 5, 10–14, 27–30 months) in the Tg2576 model, compared to an age-matched wild-type (Wt, for materials and methods see Appendix A). We have analyzed *PDGFαR*, as a marker of OPC/pre-OL stage, and *MBP* as marker for mature myelinating OLs (Figure 1). In our animal model, the mRNA expression of *PDGFαR* peaks at 5 months in both genotypes (around 6-fold in Wt and 2.5-fold in Tg2576), mRNA levels being significantly lower in the Tg2576 compared to Wt at this time point. The expression of *PDGFαR* is then down-regulated at 10–14 months and at the last group of age studied, 27–30 months, the last age group studied (Figure 1A). Overall, and according to the two-way ANOVA analysis, the expression level of *PDGFαR* is different in the two genotypes. *MBP* mRNA slightly higher in the Wt group of animals at 3 months, whereas in Tg2576 animals it decreases (around 0.5-fold), but peaks afterwards at 5 months in both genotypes (around 4-fold in Wt and 2-fold in Tg2576). It subsequently decreases at 10–14 months, finally increasing to achieve the same level of expression at the oldest age investigated (Figure 1B).

**Figure 2:**
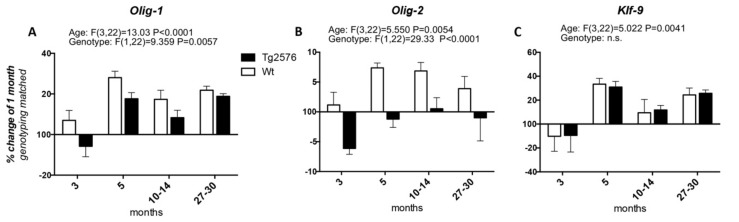
Age-related variation of oligodendrocyte precursor cells to oligodendrocyte transcription factors. The graphs show the expression profile of *Olig-1* (**A**), *Olig-2* (**B**), and *Klf-9* (**C**) transcription factors. Relative expression has been normalized to 1-month for each genotype and given the value of 100%. Results are expressed as the % of variation compared to the 1-month genotype-matched, Wt and Tg2576, groups of animals. Statistical analysis has been performed through 2-wayANOVA, considering age (months) and genotype (Wt and Tg2576) as variables; n = 3–5. Results are significant when *p* < 0.05.

We then studied the expression of the transcription factors involved in OPC differentiation into mature myelinating OLs, i.e., *Olig-1* and *Olig-2*, whose expression spans from the neuroprecursor cell stage to mature myelinating OLs, and *Klf-9* (*Krueppel-like factor 9*), which is up-regulated as soon as the OPCs exit the cell cycle in the presence of T3, to fully differentiate (Figure 2). Considering the age of 1 month as the 100%, the expression of *Olig-1* increases at 3 months of age in the Wt group of animals (at around 107%), peaks at 5 months (at around 128%), then decreases at the 10–14 and 27–30 time points (to around 117% and 121%, respectively). In the Tg2576 group of animals, *Olig-1* expression decreases at 3 months (to around 94%), increases at 5 months to the higher mRNA level (to around 117%), then decreases again at 10–14 months, finally increasing at 20–27 months, the last time point studied (at around 118%) (Figure 2A). The changes in the mRNA levels of *Olig-2* in both the Wt and Tg2576 animals are lower than the changes in *Olig-1* mRNA. In the Wt animals it reaches the highest level from between 5 and 10–14 months (at around 107%), then decreasing to 104% (Figure 2B). The *Olig-2* expression profile in the Tg2576 group of animals is the opposite of the Wt group, significantly decreasing to around 94% at 3 months, before returning to 100% at the last time points. Overall, and according to the two-way ANOVA analysis, the expression level of both *Olig-1* and *Olig-2* is different in the two genotypes. The age-related changes in *Klf-9* mRNA is the same in both Wt and Tg2576 (Figure 2C), decreasing at 3 months (to around 90%), then peaking at 5 months (to around 130%). It decreases again at 10–14 months (to around 110%) and finally it increases (to around 125%). 

To the best of our knowledge, the literature regarding the study of OPCs/OLs in AD and related animal models is scarce. Studies in APP-PS1 mice have demonstrated an increase in OPCs at 6–8 months of age [36], as observed in our Tg2576 mice where the expression level of the OPC marker *PDGFαR* gene is significantly higher at 5 months. Similarly, studies performed in postmortem human AD have revealed an increase in the number of PDGF*α*R positive cells in WM lesions [37], while a decrease in Olig-2 positive cells has been described [36]. We also observed a decrease in *Olig2* expression level at all ages in Tg2576 compared to Wt, suggesting a defect in the key molecular signaling involved in OPC differentiation in AD mice, indeed the expression of the *MBP*, the main myelin protein, is lower in the Tg2576 group than the Wt group at most ages. This hypothesis is also confirmed by the decreased number of mature non-myelinating OLs at 6 months of age in 3 × Tg-AD [38].

MBP is one of the main myelin-related protein and it is found to be associated with amyloid plaques [39]. However, different myelin proteins (i.e., MBP, MAG, MOG, and PLP) are proposed as target for early biomarkers for memory loss in AD, since the antibodies titers in sera of AD patients significantly increases in early stages of the disease [40].

## 4. OPC Vulnerability in Neurodegenerative Diseases

**Figure 3:**
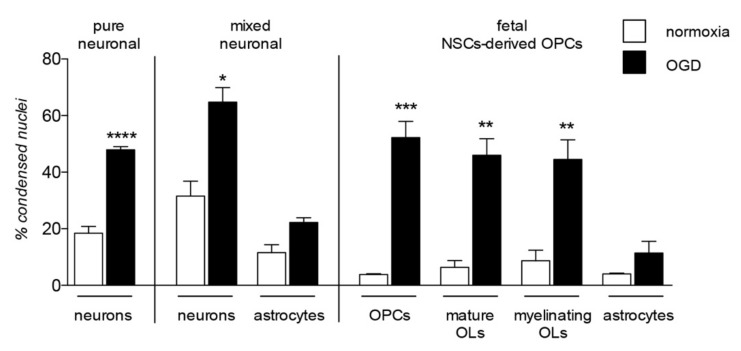
Lineage specific vulnerability to oxygen-glucose deprivation in in vitro models. The graph shows vulnerability to the in vitro model of hypoxia/ischemia, the oxygen-glucose deprivation (OGD), analyzed in three different in vitro models: pure neuronal, mixed neuronal/astroglial and neural stem cells (NSCs)-derived OPCs cultures. This is a summary chart of already published data [45,46], measured by cell-based high content screening and represented as percentage of condensed nuclei. Statistical analysis has been performed by Student’s t-test and asterisks represent the differences between normoxia and OGD exposed cells of the same lineage (* *p* < 0.05, ** *p* < 0.01, *** *p* = 0.001).

The contribution of OPC/OL injury to certain demyelinating diseases, such as MS, is clear and proven as the major cause of the neuronal degeneration. However, the damage of myelin and myelin-forming cells contribute to a wide range of neurodegenerative diseases and CNS injuries, a predictable finding given the complex role exerted by these cells in neuronal functions, not only in providing physical and anatomical support, but also as a functional player in neuronal processes, from metabolism to signaling regulation [41,42]. Cells belonging to the OL lineage are as vulnerable as neurons to noxious stimuli, enough to be considered as the most vulnerable cells of the CNS [43,44,45] highlighting their implication in the onset and progression of neurodegenerative diseases. A comparative analysis of cell death in neuronal pure and neurons-astrocytes mixed culture, in OPC at different differentiation stages (NG2-positivity for OPC, CNPase-positivity for mature OLs and MBP-positivity for myelinating OLs), and astrocytes after exposure to OGD is presented in Figure 3.

The most significant and well-described pathological mechanism affecting the cell survival and differentiation processes of the OL lineage is the establishment of an inflammatory microenvironment. It was proven that acute inflammation increases the OPCs proliferation, migration and differentiation thorough immune cells [47] and specific cytokines [48,49] as a response to demyelination insults. However, in the complex pathological environment of MS, the remyelination process progressively fails, in parallel to the establishment of the inflammation environment [50]. This is probably mediated by cytokines secreted by immune system cells as proven by the OPC differentiation block mediated by lymphocytes isolated from MS patients [51]. This leads to two main pathological outcomes: cell death of mature OLs, and OPC differentiation block, resulting in enhanced demyelination and remyelination impairment [52], which seems to be mediated by the alteration of the local content of triiodothyronine (T3) [53]. An appropriate intracellular T3 concentration is mandatory for cell cycle exit and terminal differentiation [54]. This pathological process has been widely investigated, since inflammation-mediated OPC differentiation block is considered responsible for remyelination failure in MS and other demyelinating diseases. However, the damage of myelin and myelin-forming cells is a major pathological event not only in inflammatory diseases, but also in vascular and traumatic lesions of the CNS, and a major cause of consequent neurodegeneration and chronic disabilities in both infants and adults [55]. These injuries impact on axonal integrity and function [56], making WM pathology a recognized target for pharmacological strategies [57]. Oligodendroglial lineage includes cells which are differentiating and maturing, from precursors through preOLs to myelinating OLs, showing huge differences in their biology throughout the entire differentiation process and leading to different responses to adverse stimuli [58].

The first link between the neurodegeneration described in AD and myelin cell vulnerability is the accumulation of Aβ peptide, which induces OL dysfunction and death both in vivo and in vitro [59,60]. The mechanism underlying the Aβ toxicity in OPCs and OLs is still unclear, but a number of studies seem to describe a complex multifactorial process involving different pathways: nSMase-ceramide cascade, TNFα, p75NTR, Fas, mitochondrial dysfunction, and oxidative stress, in which mature OLs seem to be more vulnerable than precursors [61]. However, a positive effect of acute exposure to Aβ oligomeric forms has also been described, increasing MBP translation in OLs, and promoting differentiation as well as cell survival in cultured OLs. The mechanism involves Itgb1 signaling, Fyn kinase and intracellular Ca^2+^ level modification. Aβ oligomers also seem to contribute to OL differentiation in cultured cerebellar slices, and to enhance remyelination following lysolecithin-induced demyelination [62]. This contrasting evidence highlights the complexity of the Aβ functions in physiological and pathological conditions, corroborating the hypothesis that OL toxicity induced by Aβ accumulation is a multifactorial process. This is also proven by the accumulation of Aβ in a mouse model of familial AD lacking the PS1 gene, showing WM dysfunctions linked to glutamate and calcium signaling dysregulation [63].

Tauopathy is the second AD hallmark, directly connected to Aβ toxicity. AD shows predominantly neuronal tau pathology in the form of neurofibrillary tangles, in contrast to other tauopathies characterized by the presence of glial tau pathologies. In OLs, this leads to myelin fragmentation and atrophy, exacerbating axonal instability. Moreover, pathological tau accumulation reduces the capability of astrocytes to uptake glutamate, leading to excitotoxicity mechanisms, an important player in both OL and neuronal vulnerability [64]. In this context, however, tau also seems to have a positive side function, in that axonal injury seems to promote the differentiation of OPCs during the remyelination process, thanks to the expression of a pathological form of tau [65].

Closely related to inflammatory pathological processes and Aβ toxicity, both OPCs and OLs are highly vulnerable to hypoxia/ischemia (HI) a common event in most neurodegenerative diseases and CNS injuries [66]. In fact, beyond the pathological mechanisms directly correlated to hypoxia and metabolic stress, HI also induces an inflammatory response [67]. Moreover, HI-induced cell death in neurons is exacerbated by Aβ accumulation [33], demonstrating how all these pathological mechanisms are interconnected. Notably, the severity and the pattern of the injury mediated by HI is strictly dependent on the developmental [68] and maturation [69] stages involved. OPCs/OLs are more vulnerable during developmental myelination, and precursors are more susceptible than mature OLs. This has been proven by the comparison between OPCs derived from fetal and from adult neural stem cells (NSCs) exposed to OGD, the in vitro model of HI. These experiments lead show that only fetal cells are vulnerable to OGD-mediated cell toxicity [45] while, notably, both fetal and adult OPCs respond in the same way to inflammation-induced differentiation block. The differing responses of fetal and adult OPCs in the HI context is not surprising, since the two cell types, even with the same derivation (i.e., the NSC) show differences in their biology and differentiation processes [70]. 

The onset of a hypoxic/ischemic microenvironment also leads to other noxious consequences, all of which undermine the cell viability and functions of both neurons and glial cells: Indeed HI affects DNA stability, induces oxidative stress, and increases iron levels [29]. An emerging hypothesis suggests myelin degradation in sporadic AD as being the one of the earliest structural changes, independent of amyloid plaque formation and linked to DNA damage [71]. Oxidative stress is also directly linked to Aβ oligomers accumulation and cell death induction [61]. In particular, OLs show a low content of glutathione and low antioxidant defenses, coupled with a high consumption of oxygen and ATP, making these cells highly vulnerable to ROS production, inducing lipid peroxidation, impairing protein and nucleic acid production and promoting membrane disruption [42,72]. Notably, this mechanism of OL degeneration seems to form the basis of the secondary degeneration in neurotrauma [73].

As mentioned above, glutamate excitotoxicity is strictly linked to Aβ toxicity, and glutamate receptor overactivation is the main player in HI-induced cell death. Glutamate exerts a physiological role in OPC differentiation and OL signaling [74], but AMPA/kainite receptors also mediate glutamate excitotoxicity in glial cells [75], and in the preOLs in particular [76]. Mature OLs especially undergo HI-induced cell death due to an early excitotoxic-oxidative cascade, caused by the reduction of the high-energy phosphate metabolism. This leads to an increase in lactic acid, and to a failure in ion transport across the cell membrane, which combined with the destruction of the cytoskeleton, causes depolarization and excessive glutamate release. This condition is also worsened by the depleted glutamate reuptake caused by the reduced availability of glucose [67]. However, due to the significant differences in response to noxious events based on the variations in their differentiation and developmental stages, glutamate receptor inhibition is unable to protect fetal NSC-derived OPCs from HI-induced cell death. In this spontaneous astrocyte/OPCs co-cultures, the glucose deprivation acts as the major trigger for HI-mediated injury. The high heterogeneity between juvenile and adult OPCs and OLs populations was also confirmed by single cell RNAseq analysis [77]. Moreover, in the adult brain transcriptome analysis revealed that OLs are not included in a single family, can be split in six different classes based on their RNA expression profile [78].

## 5. OPCs as Target for Neuroprotection

**Figure 4:**
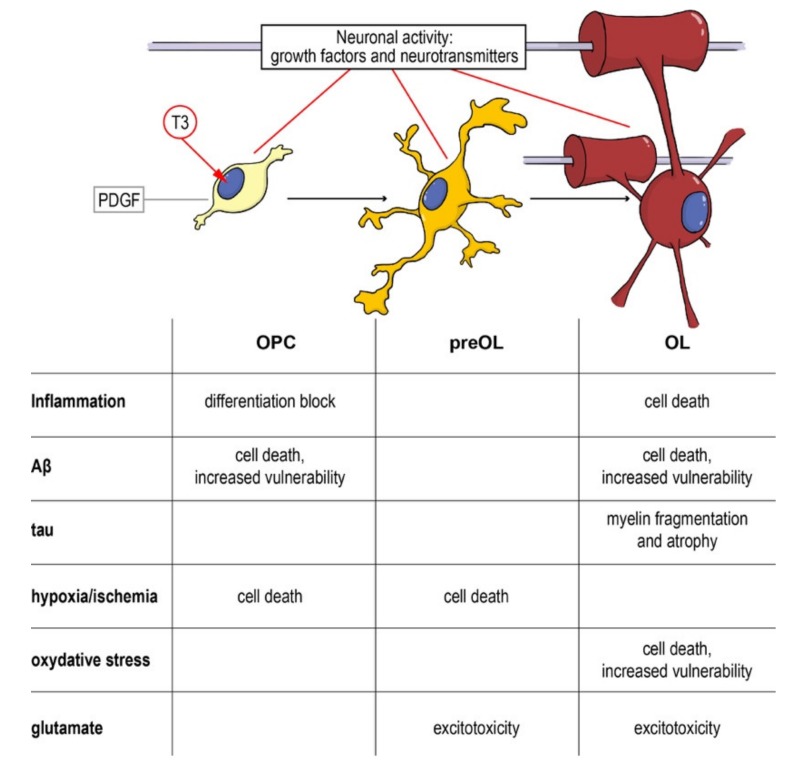
From the precursor to the mature oligodendrocyte: impact of noxious stimuli on differentiation and viability. Schematic representation of the physiological differentiation from the oligodendrocyte precursor cell (OPC) to myelinating OL. T3, the active form of the thyroid hormone, is the trigger of the process, driving the cell out of the cell cycle and starting the differentiation machinery. Neurotransmitters (such as GABA, glutamate and NO) directly contribute to regulating the process and the interaction between the axon and its activity and the OPCs/OLs. In the table are summarized the different component of neurodegenerative and demyelinating diseases affecting the different differentiation stages. Abbreviations: Aβ, amyloid beta; OL, oligodendrocyte; OPC, oligodendrocyte precursor cell; PDGF, platelet derived growth factor; T3, triiodothyronine.

As a complex and multifactorial disease, an effective neuroprotective strategy for AD should target multiple biological processes, and myelin and OPCs should be taken into account, given their fundamental role in neuronal and axonal function, and the evidences of their involvement in the disease onset and progression. Cells along the oligodendroglial lineage show remarkable differences (i.e., OPCs maturation) of physiological characteristics and vulnerability to noxious stimuli. Thus, neuroprotective strategies aimed to protect myelin and myelin forming cells should pursue two main objectives: (i) Protection of mature OLs and resident OPCs from cell death and (ii) enhancement of remyelination through proper OPCs differentiation. As described above, pathological AD mechanisms affect both of these aspects and the entire oligodendroglial lineage (Figure 4). 

The importance of myelin-forming cells in the context of neurodegeneration and neuroprotection is dramatically illustrated by demyelinating diseases such as MS. Remyelination is the most efficient regenerative process in the adult CNS, the only one that can bring about a complete anatomical and functional recovery [79] and is not performed by preexisting OLs but by resident and NSC-derived OPCs. Thus, a number of neuroprotective strategies based on OPCs protection have been suggested for MS, including restoration of proper T3 signaling impaired by inflammation [53]. It is now clear that the remyelination failure occurring in MS is a complex event involving different mechanisms, a process in which OPC differentiation failure plays a central role [80]. Strategies may directly target pathways or receptors involved in this process. and a variety of receptor tyrosine kinases and their growth factor ligands (PDGF, HGF, EGF, NGF, BDNF, and FGF), G protein-coupled receptors (histamine, muscarinic cholinergic, dopamine, and serotonin receptors) as well as other receptors [81,82,83]. The deep investigation of these physiological players in OPCs differentiation and remyelination process paves the way to a number of pharmacological trials acting on them [81]. It is also foreseeable that nuclear receptors involved in the T3 signaling may be putative targets. This is the case of RXRγ, described as one of the main partners in the thyroid hormone receptors heterodimers leading to OPCs differentiation and implied in remyelination process [84] and now clearly essential for the T3-mediated differentiation induction [70].

Screening for targets of remyelination, and molecules acting on these processes, seems to be the most widely used drug discovery strategy, based on isolated primary OPCs, embryonic/ neural stem cell-derived OPCs, or induced pluripotent cell-differentiated OPCs, all cell systems which allow a molecular analysis of the physiological OPC differentiation process [85,86]. As we are learning from MS, however, pathological conditions interfere greatly with physiological mechanisms and the microenvironment where OPCs undergo differentiation/maturation processes, highlighting the need to develop new strategies based on pathological in vitro and in vivo models [53]: One such example emerged from a pharmacological strategy developed to save neurons from cell death, based on the inhibition of the poly(ADP-ribose) polymerase (PARP). Drugs inhibiting PARP activity show promising neuroprotective action in both fetal CNS injury, such as neonatal HI, and in adult diseases, such as MS. In MS animal models, pharmacological inhibition of PARP also exerts a protective action on mature OLs [87]. However, when tested for neonatal HI, a special consideration should be given to OPCs due to of the key time window (perinatal/early postnatal) for the developmental myelination. While PARP inhibition shows no effect on adult NSCs-derived OPCs, its effects are highly toxic when performed on fetal-derived OPCs [88], again highlighting the importance of taking into account not only OPCs in neuroprotective strategies development, but also their high variability in terms of maturation and developmental stage.

Even if the fact that myelin and myelin forming cells damages may play and important role in the onset and/or progression of AD is clear, none of the AD treatment is directly targeting them. However, some treatments may indirectly target OPCs and OLs, acting on immune system and inflammation. This is the case of the above mentioned CHF5074 molecule, which is acting not only on amyloid production, but also modulating the microglia activation [89] as also proven in a phase II clinical trial [90]. 

## 6. Conclusions

The multifactorial nature of AD pathology, the multiple roles of myelin and myelin forming cells, and the accumulating evidences about WM abnormalities not only in late AD, but also in MCI, support the view that effective neuroprotective strategies should consider multiple cellular and molecular targets and specific time-windows. In this context, the capability of endogenous regeneration of myelin thanks to the presence of OPCs, offers an opportunity still to be explored.

## Appendix A

Material and Methods for RT-PCR original data reported in the manuscript.

### Appendix A1. Materials and Methods

In this study Tg2576 transgenic mice (mice carrying a transgene coding for the 695-amino acid isoform of human APP derived from a large Swedish family with early-onset AD, as described by Hsiao et al. [91] and non-transgenic littermates (001349-W, WT) were used. Animals have been purchased from Taconic Europe (Lille Skensved, Denmark).

Wild-type and Tg2576 animals at 1, 3, 5, 14, and 27-old months of age were included in the study. The number of animals for each experimental group was 3–5. Tg2576 mice were tested for the retinal degeneration Pde6b (rd1) (rd) mutation, resulting as negative.

All animal protocols described here were carried out according to the European Community Council Directives 86/609/EEC, approved by Italian Ministry of Health (D.Lgs 116/92) as well as the European Community Council Directives 2010/63/UE. Animal protocols were carried out in compliance with the guidelines published in the NIH Guide for the Care and Use of the precursor protein gene used for the experiments.

The cerebral cortex of the Wt and Tg2576 mice were processed for the isolation of total RNA using the RNAeasy Miniki (Qiagen, Milan, Italy). Isolated RNA was quantified using the Nanodrop 2000 Spectophotometer (Thermo Scientific) and then subjected to retrotranscription using the iScrip cDNA Synthesis Kit (BioRad, Hercules, CA) according to manufacturer’s instructions. To asses any possible genomic DNA contamination, no reverse transcription sample was processed in parallel as a control sample.

Semiquantitative QPCR was performed by using the SsoAdvanzed^TM^ Universal SYBR Green Supermix (BioRad). An amount of 10 ng of cDNA per sample was used for all the primer-specific QPCR reactions performed. The final volume of reactions was 20 µL in which 0.4 µM of forward and reverse primer mix was used. The control sample was also processed by QPCR for each specific primer mix used. Reactions were performed using the CFX96 QPCR instrument (BioRad) according to the following temperature/time profile: 95 °C/2 min, for polymerase activation and DNA denaturation, followed by 40 cycles of amplification (95 °C/15 s, denaturation, 60 °C/60 s, annealing/extension). Melt curve analysis of the amplified products was performed by heating from 65 °C to 95 °C with an increase of 0.5 °C/s. The specificity of the PCR reactions was revealed by the single peak obtained following melt analysis. The 2^(−∆∆Ct)^ method was used for the analysis of the relative gene expression, given that the efficiency of all the primers used herein was between 95 and 102%.

The sequence of specific primers used is described in the Table A1. The *GAPDH* was used as housekeeping gene (Cq WT vs Tg, Student’s t-test, *p* = 0.279; Cq Wt age-matched vs Tg2576 age-matched, 1-way ANOVA, *p* = 0.272).

**Table A1:** Primers sequences.

Gene	NCBI Acc. N.	Primer Sequences
*GAPDH*	NM_17701	F: 5′-ggcaagttcaatggcacagtcaag-3′ R: 5′-acatactcagcaccagcatcacc-3′
*Klf-9*	NM_010638.4	F: 5′-agtggcttcgaaggggaaac-3′ R: 5′-tccgagcgcgagaacttttt-3′
*MBP*	NM_001025251	F: 5′-gcctgtccctcagcagattt-3′ R: 5′-gtcgtaggcccccttgaatc-3′
*Olig-1*	NM_ 016968	F: 5′-ccgccccagatgtactatgc-3′ R: 5′-aacccaccagctcatacagc-3′
*Olig-2*	NM_016967.2	F: 5′-gcttagatcatccctggggc-3′ R: 5′-agatcatcgggttctgggga-3′
*PDGF* *αR*	NM_001083316.2	F: 5′-cggaacctcagagagaatcgg-3′ R: 5′-tccccatagctcctgagacc-3′

Information about gene name, NCBI (National Center for Biotechnology Information) genbank accession number, forward (F) and reverse (R) sequences description has been included. *Klf-9*,* Krueppel-like factor 9*;* GAPDH*, *Glyceraldehyde-3-phosphate dehydrogenase*;* Olig-1*, *Oligodendrocyte transcription factor 1*;* Olig-2*,* Oligodendrocyte transcription factor 2*; MBP, myelin basic protein; PDGFαR, platelet derived growth factor receptor α.
